# Vaccine Efficacy, Impact, Hesitancy, and Acceptance: Trends for Public Health

**DOI:** 10.3390/vaccines12040406

**Published:** 2024-04-11

**Authors:** Elias A. Said, Alessandra Noto, Sylvain Cardinaud, Ali A. Al-Jabri

**Affiliations:** 1 Department of Microbiology and Immunology, College of Medicine and Health Sciences, Sultan Qaboos University, P.O. Box 35, Muscat 123, Oman; aaljabri@squ.edu.om; 2 Service of Immunology and Allergy, Lausanne University Hospital, University of Lausanne, 1011 Lausanne, Switzerland; alessandra.noto@chuv.ch; 3 Vaccine Research Institute (VRI), Inserm U955-Équipe 16, Institut Mondor de Recherche Biomédicale (IMRB), Université Paris-Est Créteil (UPEC), 94010 Créteil, France; sylvain.cardinaud@inserm.fr

Vaccines are indispensable tools in the battle against infectious diseases and hold great potential in combating a myriad of other diseases. The historical achievements accomplished through vaccination, notably in eradicating smallpox and reducing the burden of numerous infections, highlight the essential role of vaccines in public health [1]. However, the success of vaccination efforts hinges not only on the efficacy of vaccines but also on societal acceptance. The willingness of populations to embrace vaccination campaigns profoundly influences their outcomes [2]. Conversely, vaccine hesitancy poses a significant challenge, potentially undermining the effectiveness of such endeavors [3]. Central to the efficacy of vaccines is the intricate interplay between the immune system and pathogens [4]. In the pursuit of effective disease prevention and eradication, the acceptance and perception of vaccines within populations play pivotal roles [2]. It is imperative to comprehend the mechanisms facilitating vaccine acceptance and their impact on disease perception among communities. Furthermore, the development of strategies aimed at enhancing vaccine acceptance is crucial for successful vaccine implementation endeavors [5]. Achieving a comprehensive approach to vaccine deployment necessitates an understanding of strategies for disease elimination through vaccination efforts [5]. Additionally, elucidating the determinants contributing to vaccine-related side effects is paramount, as such factors can significantly influence vaccine acceptance and efficacy [6]. Therefore, the articles within this Special Issue are dedicated to exploring these aspects. Four of these articles focus on vaccination considerations within the realm of coronavirus disease 2019 (COVID-19), caused by the infection with the severe acute respiratory syndrome coronavirus 2 (SARS-CoV-2), which represents a significant global threat. Additionally, the fifth article delves into vaccination parameters within the context of another prevalent global infection, hepatitis B virus (HBV) infection.

On the one hand, the systematic review and meta-analysis (SRMA) by Hajissa et al. investigated COVID-19 vaccine acceptance and hesitancy among migrants, refugees, and foreign workers (Contribution 1). The analysis of 19 peer-reviewed studies encompassing 29,152 subjects revealed an overall acceptance rate of 56.7% (95% CI: 44.9–68.5%). However, vaccine hesitancy was also prevalent, estimated at 31.7% (95% CI: 44.9–68.5%), indicating that a significant portion of this population remains skeptical about vaccination. Interestingly, the initial enthusiasm for vaccination (77.3% acceptance in 2020) declined in 2021 (52.9%) before a modest rebound in 2022 (56.1%). Concerns regarding vaccine efficacy and safety emerged as the primary drivers of hesitancy. The study underscores the need for targeted vaccination campaigns to address these concerns and improve vaccine acceptance among migrant populations, ultimately contributing to herd immunity.

On the other hand, the study by Caballero et al. describes the development and initial evaluation of a comprehensive COVID-19 vaccine education toolkit designed for community health workers (Contribution 2). The toolkit was built upon established health education practices: utilizing the Health Belief Model for content selection, employing plain language writing principles, incorporating community input, and relying on trusted messengers for dissemination. The toolkit comprised an easy-to-read workbook for community members, a leader guide with scripting for health workers, and additional resources. Notably, content development involved collaboration between plain language writers, clinicians, and subject matter experts, followed by iterative refinement based on community feedback. Preliminary evaluation via surveys suggested this toolkit effectively equipped community health workers for delivering scientific information about COVID-19 vaccines. Moreover, a significant portion of health workers reported that the toolkit facilitated community members’ decisions to get vaccinated. These findings support the potential of developing toolkits in promoting vaccine education and uptake.

Moreover, a cross-sectional study performed in Riyadh, Saudi Arabia (June-December 2021) by Al-Shouli et al. evaluated risk perception of COVID-19 among vaccinated individuals (aged 18 and above) using a questionnaire-based approach (Contribution 3). Despite the ongoing risk of infection and transmission after vaccination, 30.2% of participants maintained a high-risk perception and continued consistent protective behaviors. The study identified significant associations between risk perception and various sociodemographic factors (age, gender, marital status, occupation, employment, and income). These findings suggest that vaccination alone may not significantly alter risk perception for all individuals, highlighting the importance of considering sociodemographic factors in public health messaging to encourage continued protective behaviors.

Another cross-sectional study by Said et al. investigated factors influencing the severity of side effects following COVID-19 vaccination, especially with the BNT162b2 (Pfizer) or ChAdOx1-S (AstraZeneca) vaccines (Contribution 4). The study found that females, individuals with chronic diseases, those taking medications regularly, and those with a history of SARS-CoV-2 infection experienced more severe side effects after the second dose of BNT162b2. For the ChAdOx1-S vaccine, females and those with a history of allergies reported stronger side effects after the second dose. This study highlights the potential impact of sex, chronic conditions, medication use, allergy history, and COVID-19 infection on the severity of side effects following anti-COVID-19 vaccination with BNT162b2 and ChAdOx1-S vaccines.

Finally, a comprehensive review by Al-Busafi and Alwassief examines global trends in hepatitis B virus (HBV) vaccination coverage, assessing the profound impact of vaccination on HBV prevalence and its consequences across diverse populations, including high-risk and general demographics. It highlights the ongoing challenge of HBV infection, with over 1.5 million new cases and a global burden of 296 million chronically infected individuals, which raises significant public health concerns (Contribution 5). Chronic HBV leads to significant mortality (over 820,000 deaths annually) from complications like liver cirrhosis and hepatocellular carcinoma (HCC). HBV vaccination is the cornerstone of public health strategy to prevent chronic infection and its sequelae. The World Health Organization (WHO) has established ambitious goals for HBV elimination, including 90% global birth dose coverage by 2030. However, current coverage remains low at only 46%, highlighting a significant gap. Therefore, long-term commitment is key for maintaining progress against HBV. This requires continuous innovation, national policies, and focused elimination efforts with close monitoring of program impact.

Collectively, these studies provide valuable insights for optimizing vaccination strategies and underscore the importance of ongoing efforts to address vaccine hesitancy, educate communities, and improve vaccine coverage against diseases. Therefore, they contribute to our understanding of vaccine acceptance, education, risk perception, and side effects, providing valuable insights for public health strategies and interventions aimed at combating infections on a global scale.
